# Correlation between Hyperalgesia and Upregulation of TNF-*α* and IL-1*β* in Aqueous Humor and Blood in Second Eye Phacoemulsification: Clinical and Experimental Investigation

**DOI:** 10.1155/2021/7377685

**Published:** 2021-08-25

**Authors:** Ruibo Yang, Chang Liu, Di Yu, Lechong Ma, Yan Zhang, Shaozhen Zhao

**Affiliations:** ^1^Tianjin Key Laboratory of Retinal Functions and Diseases, Tianjin Branch of National Clinical Research Center for Ocular Disease, Eye Institute and School of Optometry, Tianjin Medical University Eye Hospital, No. 251, FuKang Road, Nankai District, Tianjin 30384, China; ^2^University of California Santa Cruz, 1156 High Street, Santa Cruz, CA 95064, USA

## Abstract

The aim of this study was to explore the correlation between intraoperative hyperalgesia of the second eye and the dynamic changes of tumor necrosis factor (TNF)-*α* and interleukin (IL)-1*β* levels in aqueous humor (AH) of the second eye and whole blood after the first eye cataract surgery. A rabbit model of monocular phacoemulsification was established by administration of 0.3% levofloxacin. Whole blood and AH samples from non-surgical eyes in the experimental group (n =25) and second eye in the blank control group (n =15) were obtained and corneal sensitivity was examined after surgery (1, 3, 7, 14, and 21 days postoperatively). TNF-*α* and IL-1*β* levels in AH and TNF-*α* mRNA and IL-1*β* mRNA levels in whole blood were measured. In a clinical study, 30 patients who underwent bilateral phacoemulsification within 1 month were divided into six groups in accordance with the operation intervals (1, 3, 7, 10, 14, and 21days). TNF-*α* and IL-1*β* levels in AH were measured at the beginning of surgery and intraoperative pain was assessed immediately after surgery. Corneal sensitivity (F =244.910, P <0.05), TNF-*α* and IL-1*β* levels in AH (F =184.200, 82.900, P <0.05) of non-surgical eyes and in whole blood (F =272.800, 193.530, P <0.05) in the experimental group were significantly higher than the baseline levels after phacoemulsification. In the clinical study, NRS scores of second eye surgery were higher than those of the first eye(P =0.0025) and 19 (63.3%) patients reported more pain during the second eye surgery. TNF-*α* and IL-1*β* concentrations in AH of the second eye were significantly higher than those of the first eye (F =123.60, P <0.05; F =59.60, P <0.05). In conclusion, within 1 month after the first eye phacoemulsification, higher pain sensitivity (hyperalgesia) exists in the second eye, which may be related to dynamic changes in TNF-*α*, IL-1*β* levels in AH or whole blood.

## 1. Introduction

Phacoemulsification and intraocular lens implantation surgery under topical anesthesia is widely performed worldwide. Because of the inconsistent visual function of both eyes, many patients undergo second eye surgery a short time after the first eye surgery [1–3]. However, more eye pain and discomfort are commonly reported by patients with bilateral cataract during the second eye surgery, despite the same surgeon, anesthetic mode, procedure, and even operation circumstances, which is considered to be related to hyperalgesia [4–9]. Intraoperative hyperalgesia may reduce a patient's willingness to cooperate, increase surgical difficulty and risk, and reduce patient postoperative satisfaction. However, the mechanism by which the pain experience is stronger during the second eye phacoemulsification is not entirely understood.

In recent years, it has been verified that tumor necrosis factor (TNF)-*α* and interleukin (IL)-1*β* not only induce leukocyte activation, chemotaxis, and inflammatory responses, but also act as neuromodulators in the nervous system to enhance the sensitivity of sensory neurons, which will lead to neuropathic pain that includes hyperalgesia [10, 11].

In the current study, TNF-*α*, IL-1*β* levels in aqueous humor (AH) of non-surgical eyes and TNF-*α* and IL-1*β* mRNA levels in blood were measured and compared with baseline levels in the phacoemulsification rabbit model. Subsequently, the TNF-*α*, IL-1*β* levels in AH were further verified between the first and second eye in clinical phacoemulsification as well as pain assessment between both eyes. The purpose of this study was to explore the correlation between intraoperative hyperalgesia of the second eye and dynamic changes of TNF-*α* and IL-1*β* levels in AH of the second eye and in whole blood after the first eye cataract surgery. To our knowledge, studies on this subject remain insufficient. Therefore, the findings from the current study may broaden our understanding on the mechanism of hyperalgesia in the second eye surgery.

## 2. Materials and Methods

### 2.1. Experimental Study

Animal experiments were approved by the Ethics Committee for Animal Studies at Tianjin Medical Eye Hospital and were in accordance with the Guide for the Care and Use of Laboratory Animals of the National Institutes of Health.

#### 2.1.1. Animals and Grouping

Forty New Zealand white rabbits (2 − 2.5 kg; Beijing Vital River Laboratory Animal Technology Co., Ltd. Beijing, China) were randomly divided into two experimental groups (group A, n =25, monocular phacoemulsification) and blank control group (group B, n =15). The rabbits were equally divided into five subgroups: postoperative 1, 3, 7, 14, and 21 day groups. Surgical eyes in group A and first eyes in group B were selected in accordance with a random number table. All animals were maintained under a 12-h light−dark cycle at 25 ± 1°C with 55% −75% humidity.

#### 2.1.2. Experimental Procedure

Rabbits were administered 0.3% levofloxacin (Santen Pharmaceutical Co, Ltd, Tokyo, Japan) four times daily for 3 days prior to the surgery. AH samples were obtained through a lateral corneal incision at the beginning of surgery in surgical eyes of group A. Surgeries were performed by the same surgeon under topical anesthesia using 0.5% proparacaine combined with intramuscular anesthetization (Xylazin Hydrochloride Injection, 0.3 mL▪kg^−1^, ShengDa Animal Medicine Company, DunHua, China). Topical Tobradex eye drops (0.3% tobramycin and 0.1% dexamethasone; Alcon Laboratories, Inc, Fort Worth, TX, USA) were administered four times a day postoperatively and tapered off for 1 month after surgery. Conjunctival congestion, corneal edema, and reaction in the anterior chamber were recorded preoperatively as well as postoperatively on days 1, 3, 7, 14, and 21. To measure baseline levels of TNF-*α* and IL-1*β* in AH and whole blood, AH of surgical eyes in group A and first eyes in group B and blood in both groups were obtained preoperatively and corneal sensitivity of non-surgical eyes in group A and second eyes in group B was assessed preoperatively. Only the surgical eyes (25 eyes) in group A were subjected to unilateral phacoemulsification uneventfully. Corneal sensitivity and inflammation cytokines (TNF-*α* and IL-1*β*) in AH of non-surgical eyes in group A and the second eyes in group, and TNF-*α* and IL-1*β* levels in whole blood of both groups were analyzed on days 1, 3, 7, 14, and 21 postoperatively. AH and blood samples from both groups were obtained and stored at −80°C. TNF-*α* and IL-1*β* levels in AH were measured by ELISAs. TNF-*α* and IL-1*β* mRNA levels in blood were measured by real-time quantitative PCR (qPCR).

#### 2.1.3. Measurement of Corneal Sensitivity

Corneal sensitivity was measured using a Cochet–Bonnet esthesiometer (Luneau Ophthalmologie, Chartres, France) after the rabbits were in good mental state and placed in a quiet indoor environment. Blinking and escape behavior were considered a positive response. Measurement by the esthesiometer started with a length of 60 mm nylon filament that was gently applied perpendicularly to the central surface of the cornea. The length of filament was shortened in steps of 5 mm until a positive response of the rabbit was observed, which was considered a positive result of corneal sensitivity. Corneal sensitivity is presented as the length of the nylon filament (mm); as the length of the nylon filament increases, the corneal sensitivity increases. The same procedure was repeated three times with a 30-minute interval; then, an average value of corneal sensitivity was taken.

#### 2.1.4. Quantitative Real-Time Polymerase Chain Reaction

Expression of TNF-*α* and IL-1*β* mRNA in blood was measured by qPCR using the ABI 7900 System (CA, USA). The primer sequences were as follows. GAPDH: sense, 5′-GATGCTGGTGCCGAGTAC-3′ and antisense 5′-GCTGAGATGATGACCCTT-3′; TNF-*α*: sense, 5′-ATGAGCACTGAAAGCAT-3′ and antisense 5′-GAGGGCTGATTAGAGAG-3′; IL-1*β*: sense, 5′-GTCTTCCTAAAGCAAGCC-3′ and antisense 5′- GGGGTGTCACAATCTGTT-3′.

#### 2.1.5. Enzyme-Linked Immunosorbent Assays

Total proteins were extracted from AH of rabbits. The concentrations of TNF-*α* and IL-1*β* in AH were measured by Enzyme-linked immunosorbent assay (ELISA) kits (TNF-*α*, Bio-Swamp Company, Wuhan, China; IL-1*β*, Bio-Swamp Company) in accordance with the manufacturer's procedures. Absorbance was read with a microplate reader (Tecan, Switzerland) at 450 nm at a reference wavelength of 570 nm. The procedure was the same for clinical samples.

### 2.2. Clinical Study

This prospective, single-blind, randomized study was conducted at Tianjin Medical University Eye Hospital between June 2018 and October 2019, which was approved by the institutional ethics committee of Tianjin Medical University Eye Hospital and followed the principles of the Declaration of Helsinki. Written informed consent was obtained from all patients. The study was registered at http://www.clinicaltrials.gov/ (registration number: ChiCTR1900023178).

#### 2.2.1. Inclusion and Exclusion Criteria

Thirty age-related cataract patients with bilateral similar grades of lens opacity and willingness to undergo bilateral cataract surgery within 1 month were recruited. Patients with any of the following were excluded: history of previous ocular surgery and other ocular diseases, and systemic diseases such as diabetes mellitus, immune diseases, and other concomitant diseases. Patients who had systemic medication history, such as sedatives, analgesics, anti-inflammatory drugs or steroids, were also excluded.

#### 2.2.2. Surgical Procedures and Grouping

The first eye was selected randomly. The timing of the second eye surgery was determined in accordance with the recovery of the first eye and the patients' willingness. The patients were divided into six groups in accordance with the interval between the two eye surgeries (1, 3, 7, 10, 14, and 21 days). Bilateral surgeries were performed by the same surgeon sequentially and uneventfully within 1 month with the same anesthetic mode (topical anesthesia, proparacaine 0.5%; Alcon, Inc, Fort Worth, TX, USA), procedures, phaco machine (Infiniti, Alcon Laboratories, Incorporated, Fort Worth, TX, USA), and operation circumstances. All surgeries were carried out between 9 and 12 am. Neither oral nor intravenous sedatives or analgesics were administered. AH samples were collected from separate single eye operations, which were obtained at the beginning of the phacoemulsification through an assisted corneal incision by inserting a 27 G needle into the anterior chamber. The samples were stored at −80°C until cytokine analysis.

#### 2.2.3. Assessment of Subjective Symptoms

Intraoperative pain was assessed at the end of the surgery using a numerical rating scale (NRS) from 0 to 10 where 0, 1 − 3, 4 − 6, and 7 − 10 indicated no pain, mild pain, moderate pain, and severe pain, respectively. After second eye surgery patients were asked if they felt more pain during the first or second surgery, assumed answers were “I had more pain during the first surgery”, “I had more pain during the second surgery”, “I experienced the same pain during both surgeries”, and “I forgot”.

#### 2.2.4. Analysis of Cytokines in AH

The procedure was the same as described for the experimental study.

### 2.3. Statistical Analysis

SPSS software version 20.0 (IBM-SPSS, Armonk, NY, USA) was used for all statistical analyses. The chi-squared test was used to determine subjective experience and NRS scores were subjected to the Wilcoxon matched-pairs signed-ranks test in the clinical study. Repeated measures analysis of variance (ANOVA) and a post-hoc LSD test were used to assess the differences in corneal sensitivity as well as TNF-*α* and IL-1*β* in blood and AH. P <0.05 was considered statistically significant.

## 3. Results

### 3.1. Experimental Study

#### 3.1.1. Corneal Sensitivity

**Figure 1:**
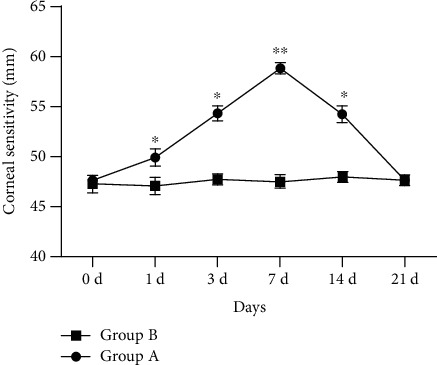
Corneal sensitivity of non-surgical eyes in group A and second-eye in group B. Group A: monocular phacoemulsification; Group B: blank control.

The corneal sensitivity of non-surgical eyes in group A was significantly higher than baseline at 1, 3, 7, and 14 days after surgery (F =244.910, P <0.05), particularly on day 7 after surgery (58.53 ± 0.38 mm), which decreased gradually and reached the baseline level at 21 days after surgery (49.60 ± 0.68 mm). There was no statistical difference between the various time points in group B (F =1.344, P >0.05). (Figure 1).

#### 3.1.2. Expressions of TNF-*α* and IL-1*β* mRNA in Blood

**Figure 2:**
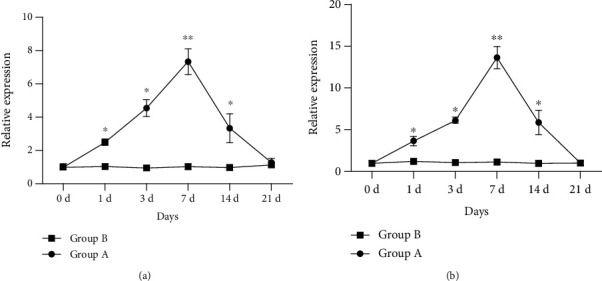
Expression of TNF-*α* mRNA and IL-1*β* mRNAs in blood of experimental rabbits. Group A: monocular phacoemulsification; Group B: blank control.

The expression of TNF-*α* and IL-1*β* mRNA in blood was significantly higher at 1, 3, 7, and 14 days after surgery compared with baseline levels in the experimental groups (*F* =272.800, P <0.05; F =193.530, P <0.05), which reached a peak on day 7 postoperatively (TNF-*α* mRNA: 14.95 ± 0.89; IL-1*β* mRNA: 7.56 ± 0.46) and then decreased gradually. TNF-*α* and IL-1*β* mRNAs returned to baseline levels on day 21 after surgery (TNF-*α* mRNA: 1.29 ± 0.27; IL-1*β* mRNA: 1.03 ± 0.20). Statistical analysis demonstrated no significant differences in the second eye at various time points in the blank control group (F =1.612, P >0.05; F =0.626, P >0.05). (Figure 2).

#### 3.1.3. Concentrations of TNF-*α* and IL-1*β* in AH

**Figure 3:**
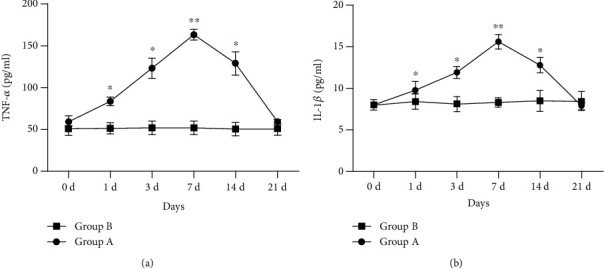
Concentrations of TNF-*α* and IL-1*β* in AH of experimental rabbits. Group A: monocular phacoemulsification; Group B: blank control.

The concentrations of TNF-*α* and IL-1*β* in AH of the non-surgical eye were significantly higher at 1, 3, 7, and 14 days after surgery than baseline levels in experimental groups (F =184.200, P <0.05; F =82.900, P <0.05), which reached a peak on day 7 postoperatively (TNF-*α*: 162.34 ± 5.71 pg/ml; IL-1*β*: 16.68 ± 0.74 pg/ml) and then decreased gradually. TNF-*α* and IL-1*β* concentrations returned to baseline levels on day 21 after surgery (TNF-*α*: 58.37 ± 9.46 pg/ml; IL-1*β*: 9.64 ± 0.75 pg/ml). Statistical analysis demonstrated no significant differences in the second eye at the virous time points in the blank control group (F =1.140, P >0.05; F =0.820, P >0.05) (Figure 3).

### 3.2. Clinical Study

A total of 30 patients (60 eyes) were enrolled in the study. The average age was 72.2 ± 8.4 years. The average duration of first eye surgery was 11.5 ± 1.9 min in the first eye, and 11.7 ± 1.8 min in second eye surgery. No significant difference was found between them (P >0.05).

#### 3.2.1. Evaluation of Eye Pain

**Figure 4:**
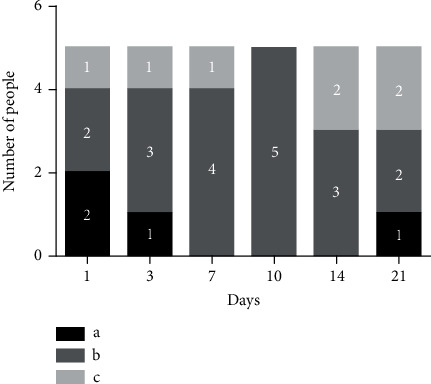
Comparison of pain experiences between bilateral surgeries. The days represent the various groups in accordance with the interval between bilateral surgeries. a: “I had more pain during the first surgery”; b: “I had more pain during the second surgery”; c: “I experienced the same pain during both surgeries”. The bar graph shows the number of people who chose the option.

Using the NRS, eye pain was significantly higher in patients who underwent second eye surgery (t =4.568, p =0.0025), particularly when the interval between the two surgeries was 10 days (p =0.016) (Table 1). By comparing pain experiences between bilateral surgeries, 19 (63.3%) patients reported more pain during the second eye surgery, seven (23.3%) patients reported the same pain during the two surgeries, four (13.3%) patients reported more pain during the first eye surgery (*χ*^2^ = 9.586, P >0.05) (Figure 4).

#### 3.2.2. Concentrations of TNF-*α* and IL-1*β* in AH

**Figure 5:**
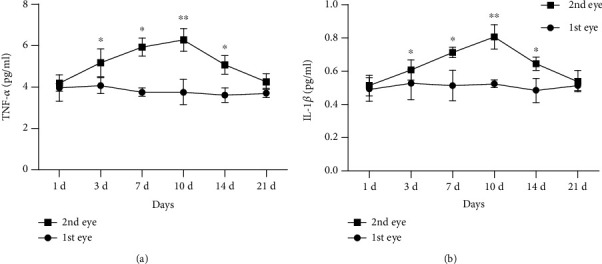
Concentrations of TNF-*α* and IL-1*β* in AH of both eyes during bilateral cataract surgery.

The concentrations of TNF-*α* and IL-1*β* were significantly higher in the second eye than the first eye in each group (F =123.60, P <0.05; F =59.60, P <0.05). Particularly at the 10-day interval, TNF-*α* and IL-1*β* levels (6.88 ± 0.71 and 0.79 ± 0.14 pg/ml, respectively) were the highest in the second eye (Figure 5).

## 4. Discussion

Under topical anesthesia, it is common that patients complain more about pain and discomfort during the second eye cataract surgery [4–9, 12–14]. Intraoperative pain may increase surgical difficulty and risk, and cause patient postoperative dissatisfaction. However, the mechanism is not yet fully understood.

Previous studies have found that cataract patients are likely to have more pain during second eye surgery, which may be associated with decreased preoperative anxiety or the amnestic effects of intravenous sedation [5, 12]. Ursea et al. proposed the following three possible mechanisms. A psychological effect would be that patients are not as well prepared for the degree of wakefulness and magnitude of sensation that they will experience during the procedure in the second eye operation. A physiological response would be that sympathetic irritation sensitizes the second eye to painful stimuli after first eye surgery. A pharmacological explanation would be that the response to analgesic and sedative medications will decreased during the second operation because of previous exposure to those same medications during the first surgical procedure, which might result in drug tolerance [15]. Nevertheless, most previous studies have mainly focused on subjective psychological mechanisms and none of these hypotheses have been proven to date. Additionally, in some studies, local anesthetic decreases together with intravenous sedation and analgesics are applied commonly during the surgery [8, 12], and eye pain has been assessed using only self-report questionnaires [4, 5, 7, 12], which would result in bias in the pain experience of patients. We speculate that higher sensitivity to pain is not merely a psychological phenomenon and the involvement of physiopathological mechanism is possible.

Neuropathic pain is associated with chronic inflammation. The mechanism that underlies the role of TNF-*α* in the development of inflammatory hyperalgesia has been studied extensively, but mainly its role in the release of proinflammatory cytokines [16–19]. In general, TNF-*α* and IL-1*β* are weekly expressed in central and peripheral nervous systems, but cytokine expression upregulates after injury. TNF-*α* stimulates cascade release of other proinflammatory cytokines, such as IL-1*β*, interleukin-6 and interleukin-8, which triggers the release of the final inflammatory mediator prostaglandin E2 and sympathetic amines that directly sensitize nociceptors [20]. In models of neuropathic pain, these cytokines activate in the nervous tissue consistent with the emergence of painful behavior, namely allodynia and hyperalgesia. Verri Jr et al. [21] suggested that Granulocyte-colony stimulating factor-induced hyperalgesia might be mediated by the peripheral production of pronociceptive cytokines TNF-*α* and IL-1*β*. Cataract surgery is an invasive procedure that breaks the blood-aqueous humor barrier, which leads to an intraocular inflammatory response in the surgical eye. A previous study revealed that AH of the second eye has higher levels of TGF-*β*2 and MCP-1 during bilateral sequential cataract surgery [22–24, 25]. CCI is produced by loose ligation of the sciatic nerve in rats and the paw withdrawal threshold to mechanical stimulation is used to evaluate pain threshold changes. We indirectly evaluated changes in eye pain perception by measuring corneal sensitivity in rabbits by observing eye blinks and escape behaviors after mechanical stimulation. The results showed that the changes of corneal sensitivity in rabbits coincided with changes of TNF-*α* and IL-1*β* levels in AH and blood, which implies that intraoperative hyperalgesia is associated with the relative high levels of TNF-*α* and IL-1*β* in the second eye AH and whole blood after first eye cataract surgery. The high levels of TNF-*α* and IL-1*β* in AH and blood will probably lead to sensitization of the sensory branches of the trigeminal nerve, which will in turn lead to hyperalgesia of the eye. Therefore, we speculate that the increased sensitivity to pain in the second eye after the first cataract surgery is not just a simple psychological response, but a complex pathophysiological mechanism.

In this study, the increased sensitivity to pain in the second eye was manifested consistently in both experimental and clinical studies, which was particularly obvious during the early postoperative period (in 2 weeks after the first eye surgery). This finding suggests that the second eye cataract surgery should be scheduled more reasonably to avoid or reduce patient discomfort and complaints. In addition to visual function, patient comfort is a crucial part of the treatment experience. Nonsteroidal anti-inflammatory drugs or analgesics may be an appropriate choice for individuals who need a second eye operation within 1 month, especially within 7–10 days of the first eye surgery.

The current study had some unavoidable limitations that need to be considered. The expression of cytokines in whole blood samples of patients was not detected because such invasive practices would inflict additional pain on the patients. Because of the amount of AH samples, we could not evaluate more cytokines related to pain perception and inflammation.

## 5. Conclusions

In conclusion, within 1 month after the first eye phacoemulsification, higher pain sensitivity (hyperalgesia) exists in the second eye, which may be related to dynamic changes of TNF-*α*, IL-1*β* levels in AH and blood. The increased inflammatory cytokines levels in AH of the second eye or non-surgical eye and blood may be induced by the first eye surgical trauma. However, further studies should be conducted to verify the regulatory pathways responsible for the increased levels of TNF-*α* and IL-1*β* in body fluids and the higher corneal sensitivity and intraoperative hyperalgesia of the second eye.

## Figures and Tables

**Table 1:** Comparison of NRS scores during bilateral cataract surgery.

	1d	3d	7d	10d	14d	21d	Total
1^st^-eye surgery	1.90 ± 0.74	2.10 ± 0.65	2.10 ± 0.74	1.90 ± 0.42	1.40 ± 0.55	2.10 ± 0.58	1.88 ± 0.12
2^nd^-eye surgery	2.10 ± 0.65	2.30 ± 0.57	2.50 ± 0.50	2.30 ± 0.45	2.50 ± 0.50	2.50 ± 0.44	2.37 ± 0.10
*P* value	0.178	0.178	0.099	0.016^∗^	0.051	0.099	0.0025^∗^

Note: 1d, 3d, 7d, 10d, 14d, 21d represent the intervals between bilateral surgeries.

## Data Availability

The datasets used in this study are available from the corresponding author on resonable request.
